# An athymic rat model of cutaneous radiation injury designed to study human tissue-based wound therapy

**DOI:** 10.1186/1748-717X-7-68

**Published:** 2012-05-08

**Authors:** Lucas H Rifkin, Strahinja Stojadinovic, Collin H Stewart, Kwang H Song, Michael C Maxted, Marcus H Bell, Natalie S Kashefi, Michael P Speiser, Michel Saint-Cyr, Michael D Story, Rod J Rohrich, Spencer A Brown, Timothy D Solberg

**Affiliations:** 1 Department of Plastic and Reconstructive Surgery, University of Texas Southwestern Medical Center, Dallas, TX, USA; 2 Division of Medical Physics and Engineering, Department of Radiation Oncology, University of Texas Southwestern Medical Center, Dallas, TX, USA; 3 Division of Molecular Radiation Biology, Department of Radiation Oncology, University of Texas Southwestern Medical Center, Dallas, TX, USA

**Keywords:** Acute cutaneous radiation injury, Normal tissue toxicity, Kilovoltage x-ray irradiation, Immunodeficient athymic rat model, Adipose-derived stem cell

## Abstract

**Purpose:**

To describe a pilot study for a novel preclinical model used to test human tissue-based therapies in the setting of cutaneous radiation injury.

**Methods:**

A protocol was designed to irradiate the skin of athymic rats while sparing the body and internal organs by utilizing a non-occlusive skin clamp along with an x-ray image guided stereotactic irradiator. Each rat was irradiated both on the right and the left flank with a circular field at a 20 cm source-to-surface distance (SSD). Single fractions of 30.4 Gy, 41.5 Gy, 52.6 Gy, 65.5 Gy, and 76.5 Gy were applied in a dose-finding trial. Eight additional wounds were created using the 41.5 Gy dose level. Each wound was photographed and the percentage of the irradiated area ulcerated at given time points was analyzed using ImageJ software.

**Results:**

No systemic or lethal sequelae occurred in any animals, and all irradiated skin areas in the multi-dose trial underwent ulceration. Greater than 60% of skin within each irradiated zone underwent ulceration within ten days, with peak ulceration ranging from 62.1% to 79.8%. Peak ulceration showed a weak correlation with radiation dose (r = 0.664). Mean ulceration rate over the study period is more closely correlated to dose (r = 0.753). With the highest dose excluded due to contraction-related distortions, correlation between dose and average ulceration showed a stronger relationship (r = 0.895). Eight additional wounds created using 41.5 Gy all reached peak ulceration above 50%, with all healing significantly but incompletely by the 65-day endpoint.

**Conclusions:**

We developed a functional preclinical model which is currently used to evaluate human tissue-based therapies in the setting of cutaneous radiation injury. Similar models may be widely applicable and useful the development of novel therapies which may improve radiotherapy management over a broad clinical spectrum.

## Introduction

Radiation is an essential modality in the treatment of malignancy, with over 60% of cancer patients receiving radiotherapy. Advances in radiotherapy have improved outcomes and resulted in higher rates of local control, contributing to a 13.6% overall reduction in cancer death rates between 1991 and 2004 [1]. Effective radiotherapy represents a dynamic balance between maximizing tumor control and sparing of healthy tissue. Nevertheless, treatment-resistant malignancies may demand aggressive radiotherapy despite an increased risk of normal tissue toxicity. In contemporary external beam radiotherapy, the use of megavoltage photon energies allows the majority of a dose to be delivered below the skin, subjecting tumors to high levels of radiation while minimizing cutaneous damage. However, skin-sparing may be reduced or even lost due to oblique beam angles, carbon fiber tables, or contamination of the beam with electrons and low-energy photons. Clinicians must carefully consider the properties of radiotherapy modalities because they impact the skin response, as outcomes of radiotherapy are often determined by characterizing the severity and the onset of radiation skin toxicity. Skin can be a dose-limiting tissue for certain cancer patient populations, such as tumors of the breast, head, and neck. In these sites, cutaneous radiation injury is one of the major concerns. Close proximity of skin to the surgical cavity often excludes patients from pursuing brachytherapy treatments such as accelerated partial breast irradiation (APBI) using balloon applicators. Recently, a prospective clinical study to evaluate APBI was closed early due to cutaneous injury [2]. Although treatment plans adhered to dosimetric requirements of the national APBI trial, 7 out of 34 patients developed new and unacceptable cosmetic outcomes.

In general, the human skin response to ionizing radiation is highly complex and dependent on the conditions of the exposure [3]. Early effects are characterized by damage to the epidermis, while late effects arise from insult to the dermal vasculature. Acute changes begin within hours as a transient erythema which subsides after 1 to 2 days, while a more intense erythematous reaction follows. Within 3 to 6 weeks, dry and moist desquamation may occur with a secondary ulceration possible 6 weeks or longer thereafter. Between 8 and 16 weeks, dermal ischemia and dermal necrosis may result in another erythematous phase. Late skin damage begins with dermal atrophy as early as 6 months, with telangiectasia and invasive fibrosis following after 1 year or longer. In clinical radiotherapy practice, skin necrosis and telangiectasia are two endpoints used to maintain the standard of care, with a 5-year 50% complication rate estimated to occur at doses of 65 Gy for telangiectasia and 70 Gy for necrosis [4]. The presentation of radiation-induced skin damage varies across animal models, but the underlying mechanism of injury and pathologic changes are similar to human tissue. In animal models there is a plethora of data on skin tolerance, largely from the era predating medical accelerators using megavoltage energies [5-16].

Current treatments and research [17,18] for cutaneous radiation damage are limited, but future discoveries may provide therapies which revitalize affected tissue and ameliorate the progressive deterioration of skin. This publication describes the design of a novel protocol to apply x-ray radiation to the skin of athymic rats. A multi-dose trial is followed by more extensive testing of a single dose. The end result is a functional preclinical model which is now used to test human tissue-based therapies in the setting of cutaneous radiation injury. Such treatments could be widely applicable, easily administered, and could significantly improve radiotherapy management over a broad clinical spectrum.

## Materials and methods

### Image guided stereotactic irradiator

An x-ray image guided stereotactic irradiator was designed for small animal studies. The characteristics and evaluation of the image guidance system are described in more detail in other articles [19-22]. In brief, main components include a commercial x-ray device (XRAD 320, Precision X-Ray Inc., N. Branford, CT), a custom-built collimation system, and an image guidance system. The XRAD 320 has a tube current ranging from 0.1 to 45 mA and a tube potential ranging from 5 to 320 kVp, permitting both diagnostic and therapeutic applications. The collimation system is based on a 20 mm diameter collimator mounted beneath the x-ray tube. The collimator is 25.4 mm thick, effectively eliminating transmitted radiation. Copper-tungsten alloy inserts can be placed within the collimator to reduce beam diameter, but none were used in this study. The imaging system includes a high resolution x-ray intensifying screen (Kodak Min-R2, Rochester, NY) which converts x-rays reaching the screen into visible light forming an image captured by a commercial CCD camera. The imaging device resolution and contrast were sufficient to clearly display anatomic structures in a small animal. For image guidance, a tube potential of 30 kVp and tube current of 10 mA were used. Target localization began by placing the animal in the irradiator and acquiring a pre-localization image. Next, animal position was adjusted using a manual XY linear translation stage coupled to the bed. Another reference image confirmed the animal position relative to the central beam axis.

### Animal preparation and irradiation

**Figure 1:**
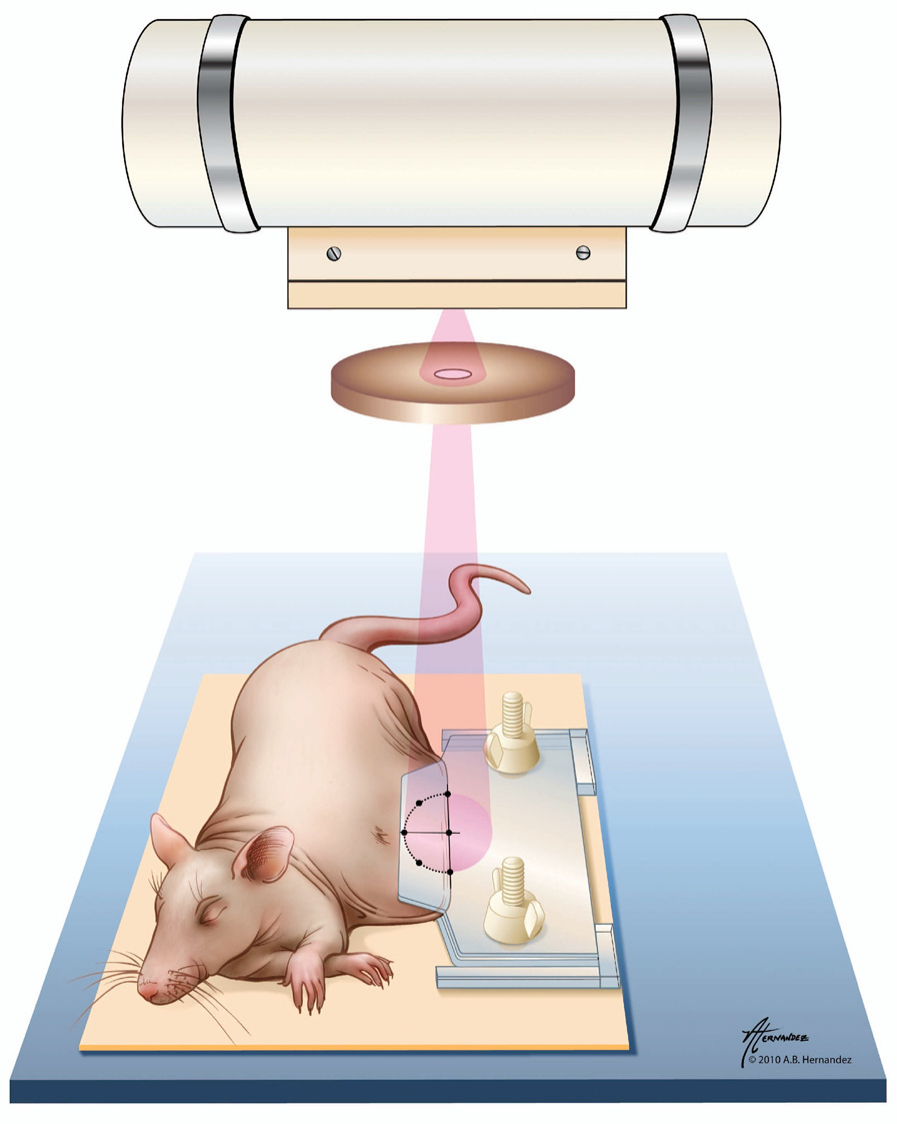
**Schematic of rat irradiation.** Under anesthesia, skin was held outward in an acrylic clamp and exposed to x-ray radiation. The beam was centered on the skin edge, traversing a semicircular skin fold. As a result, a circular area of skin was irradiated.

A protocol was designed to irradiate the skin of athymic rats while sparing the body and internal organs from significant damage, the setup is shown in Figure 1. The loose lateral skin was gently pulled away from the body and held outward in a clamp. The clamp was constructed from two acrylic sheets with the gripping surface lightly abraded to increase friction. Acrylic spacers maintained the distance between the sheets to at least 1.8 mm to prevent vascular occlusion and crush injury. The clamp contained six holes of 1 mm diameter drilled through both sheets along the border of a 20 mm diameter semicircle. The six radiolucent holes were visible throughout the image guidance process and provided outline of the skin to be irradiated. As indicated in Figure 1, the three holes forming the diameter of the semicircle were aligned with the edge of folded skin. Next, the x-ray beam was made coincident with the radiolucent holes using the image guidance system. Due to geometric divergence, the beam diameter at treatment distance was slightly larger (26.6 mm) than the diameter of the collimator (20 mm).

Female athymic rats, NIH-*Foxn1*^rnu^ strain code 316, were irradiated at eight weeks of age and at an average mass of 180 grams. This strain athymic nude rat is T-cell deficient and shows depleted cell populations in thymus-dependent areas of peripheral lymphoid organs. Each rat was irradiated twice, once on the left side and once on the right side using the same dose. Each skin site was irradiated at 250 kVp and 15 mA with the addition of 1.65 mm Al filtration to remove low energy photons from the beam at a 20 cm source-to-surface distance (SSD). The irradiator was calibrated following the AAPM Task Group 61 protocol [23], as a result the dose rate at the surface of the animals was determined to be 18.43 Gy/min with an inherent 2.9 s shutter timing error. The irradiation times were: 1.7 min, 2.3 min, 2.9 min, 3.6 min, and 4.2 min, resulting in respective doses of 30.4 Gy, 41.5 Gy, 52.6 Gy, 65.5 Gy, and 76.5 Gy. Following the multi-dose study, eight additional wounds were created using the 41.5 Gy dose. Rats were housed individually to prevent gnawing of wounds and other potentially damaging interactions.

In the multi-dose study, irradiation was followed by the placement of tattoo marks demarcating the border of the affected skin area. With the clamp still attached, ink was applied to skin by placing a needle through the semicircular pattern of holes in both sides of the clamp. A circular pattern outlining the irradiated zone was created along with an additional mark at its center. No tattooing was performed during the subsequent eight-wound 41.5 Gy experiment.

### Wound analysis

**Figure 2:**
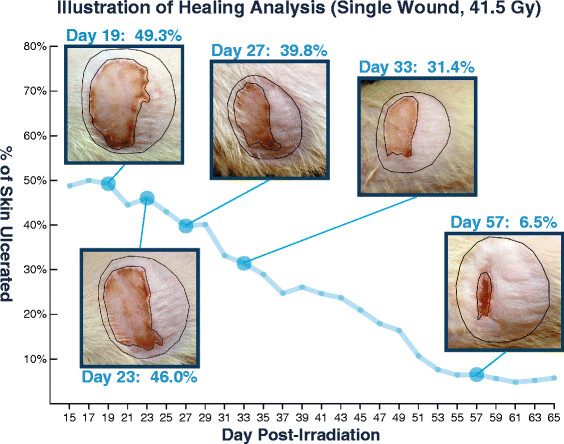
**An example of one rat wound created using a 41.5 Gy dose.** The selected images show the ulceration region within the area of irradiated skin. ImageJ software was used to calculate the ulceration percentage. All data in this figure represent the same wound at different time points.

Rats in the initial multi-dose trial were photographed on days 0 to 10 post-irradiation and on even-numbered days from 10 to 100. The eight additional 41.5 Gy wounds were photographed on each day from 0 to 15 and on each odd-numbered day from 15 to 65. Multiple images of each wound were obtained and a single best image was selected based on lighting, focus, angle, and clarity of the wound area. The percentage of the irradiated area ulcerated at each time point was determined utilizing ImageJ version 1.42 (NIH, Bethesda, MD), a public domain Java image processing and analysis software. Blinded volunteers outlined the borders of the total irradiated area and the ulcerated area, examples are shown in Figure 2. The irradiated area was distinguished from unaffected areas based on hair loss within the irradiated area and other differences in skin appearance. The ulcerated area was defined as any skin affected by scabbing, crusting, or desquamation. The number of pixels within each zone was calculated using ImageJ, and these counts were used to calculate the percentage of skin within the irradiated zone which was ulcerated at any given time. Figure 2 illustrates this methodology by plotting ulceration over time using a single example wound along with photographs corresponding to select time points.

### Animal care and euthanasia

For all procedures, anesthesia was induced using an intraperitoneal injection of ketamine, xylazine, and acepromazine at 50 mg/kg, 25 mg/kg, and 1 mg/kg, respectively. Following anesthesia, rats were placed on a heating pad and monitored until they were ambulatory. Study animals were euthanized using an intraperitoneal injection of pentobarbital at 100 mg/kg. All animal procedures were performed according to local and National Institutes of Health guidelines and approved by the University of Texas Southwestern Institutional Animal Care and Use Committee.

## Results

### Multi-dose trial

No systemic or lethal sequelae occurred in any study animals. All irradiated skin areas underwent significant ulceration, with greater than 60% of skin within each irradiated zone having ulcerated after ten days. The peak ulceration levels ranged from 62.1% to 79.8%, as shown in Table 1. Peak ulceration showed a weak correlation (r = 0.664) with radiation dose. Table 1 also shows the average ulceration percentage over the study period for each wound, which is more closely correlated to dose (r = 0.753). When the 76.5 Gy dose was excluded due to contraction-related distortion, correlation between dose and average ulceration during the study was stronger (r = 0.895).

**Table 1:** Ulceration data for five rats in the multi-dose trial, arranged by dose level.

	Peak Ulceration Percentage: Days 10-100									
	30.4 Gy		41.5 Gy		52.6 Gy		65.5 Gy		76.5 Gy	
	Left	Right	Left	Right	Left	Right	Left	Right	Left	Right
Percentage of Skin Ulcerated	75.0% Day 12	65.4% Day 10	74.4% Day 12	62.1% Day 12	78.8% Day 14	74.6% Day 12	78.9% Day 16	74.9% day 14	78.6 Day 16	79.8% Day 12
	Average Ulceration: Days 10-100									
	30.4 Gy		41.5 Gy		52.6 Gy		65.5 Gy		76.5 Gy	
	Left	Right	Left	Right	Left	Right	Left	Right	Left	Right
	19.5%	2.6%	44.8%	17.6%	42.8%	46.6%	59.8%	55.4%	37.1%	53.7%

**Figure 3:**
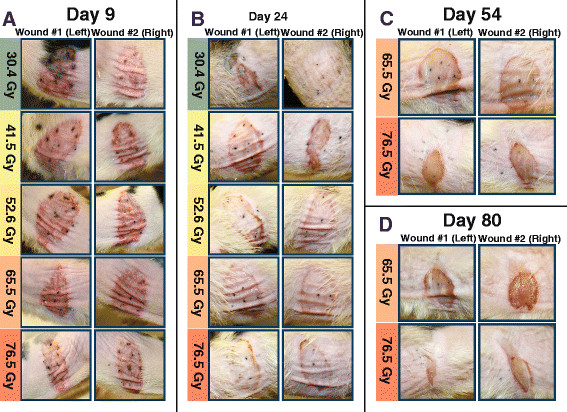
**The appearance of irradiated areas throughout the 80-day period.** The photographs are for five rats, one rat for each dose level. Each rat was wounded twice, once on the left side and once on the right side using the same dose. **A.** By day 9, erythema predominated within all irradiated areas. Crusting and desquamation were beginning to develop. Reactions intensified quickly, yet displayed a relatively uniform wound appearance across the entire dose range. Areas of increased reactivity were visible surrounding tattoo marks. **B.** By day 24, lower dose wounds (30.4 Gy and 41.5 Gy) each had a distinctly different appearance, while higher dose wounds (52.6 Gy to 76.5 Gy) exhibited features similar to other higher dose wounds. A 30.4 Gy wound had healed completely by day 20, accompanied by the return of hair growth in the irradiated area. Both 41.5 Gy wounds had undergone dry desquamation. Higher dose wounds displayed varying degrees of moist desquamation surrounded by intense crusting at the borders. **C.** By day 54, contraction in the 76.5 Gy wounds has significantly reduced the overall area of irradiated skin as well as the enclosed area of ulceration. A relatively low level of contraction was seen in the 65.5 Gy wounds. **D.** By day 80, contraction is apparent in the 65.5 Gy wounds, but impacts area and shape to a lesser degree than the higher dose. In the 76.5 Gy rat, contraction has distorted the irradiated area to such an extent that it has little resemblance to its original circular shape.

During the first ten days, wounds in all five dose groups shared a similar appearance. By day five, all affected areas clearly displayed the acute effects of erythema, mild dryness, and hair loss. By day seven, areas of intense reaction had appeared, found most prominently near tattoo marks. The eighth day was characterized by patchy scaling and increasing dryness. On the ninth day, skin reactions intensified markedly, see Figure 3A. The close resemblance among all wounds in the multi-dose trial was no longer present by day 12, as each wound in the 30.4 Gy and 41.5 Gy groups had a distinct appearance. Meanwhile, the majority of each zone irradiated with doses of 52.6 Gy, 65.5 Gy, and 76.5 Gy had fully desquamated. Through the fourth week, the higher dose (≥ 52.6 Gy) wounds shared similar features and, underwent only minimal changes in appearance. Figure 3B shows photographs of these wounds (≥ 52.6 Gy) taken on day 24, which resembles the consistent appearance seen from day 12 through day 28.

**Figure 4:**
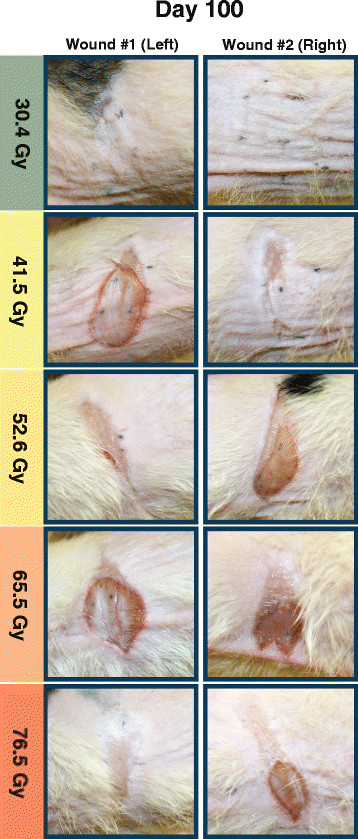
**Photographs obtained on the final day of the study for five rats, one rat for each dose level.** Significant contraction is visible in all wounds created using doses of 52.6 Gy and higher. Contraction is seen to a lesser degree on one wound (left) created using the 41.5 Gy dose. Hair growth has resumed within areas irradiated using a 30.4 Gy dose.

Wound contraction occurred within all areas irradiated with 52.6 Gy and higher doses. One additional 41.5 Gy wound underwent contraction, but only after the 80th day and to a minute degree. Neither of 30.4 Gy wounds contracted. Distortion in the shape and size of wounds ranged from minimal to severe. With increased dose, contraction began earlier and occurred with increased intensity. As contraction progressed, the dimension of irradiated areas was reduced from an anterior to posterior direction while the distance between the superior and inferior borders remained constant. Contraction began earliest and was most severe in the highest dose 76.5 Gy group. Figure 3C-3D illustrates the extreme contraction distinguishing this group from the 65.5 Gy and lower dose groups. Figure 4 presents photographs obtained on the final experimental day.

### Eight-wound validation with 41.5 Gy dose

**Figure 5:**
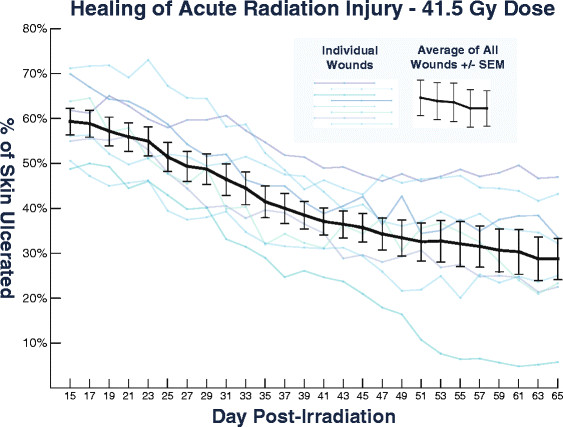
**Healing progression of eight rats, each with one wound created using a 41.5 Gy dose.** By day 65 post-irradiation, all wounds healed significantly but ulceration remained within 5.8% to 47.0% of each irradiated zone.

Following the multi-dose trial, eight additional rats received radiation dose of 41.5 Gy. Neither systemic effects nor organ damage were observed. The earliest images analyzed at day 15 had ulceration percentages ranging from 48.8% to 72.2% with a mean of 59.6% and standard error of mean (SEM) of 3.0%. No rats healed completely by the experimental endpoint at day 65. Final ulceration percentage ranged from 5.8% to 47.0% with a mean of 29.1% and SEM of 4.6%. Healing progression in this group is presented in Figure 5.

**Figure 6:**
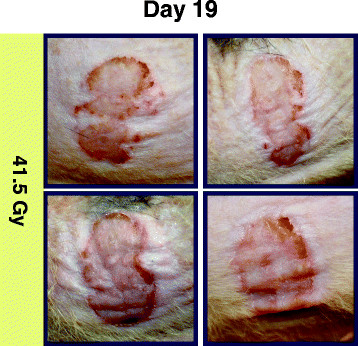
**Four rats’ wounds in the additional group irradiated with 41.5 Gy.** Appearance was similar to 41.5 Gy wounds in the multi-dose trial, without tattooing. Borders were irregular rather than maintaining a smooth, circular shape. Scabbing, crusting, and signs of healing were observed throughout wounds.

The eight wounds from the 41.5 Gy dose validation experiment and the trial wounds irradiated using the same dose displayed similarities with respect to the progression of healing, see Figure 6. All eight developed irregular margins soon after ulceration had peaked. Scabbing, crusting, and dry desquamation were observed throughout irradiated areas unlike the higher dose (≥ 52.6 Gy) trial wounds. None of the additional 41.5 Gy wounds contracted or developed moist desquamation by the 65-day endpoint.

## Discussion

The multi-dose trial established a protocol to safely and reliably deliver cutaneous x-ray radiation to athymic rats without causing systemic effects. However, the trial sample size was insufficient to rule out the possibility of adverse events within the tested range. Subsequent experiments involved eight skin areas irradiated with a 41.5 Gy dose and followed for 65 days. Ulceration occurred in the majority of each affected area and systemic damage was not observed, establishing safety of the model at this dose. A 41.5 Gy dose was selected because it produced moderate skin damage with minimal early contraction. Furthermore, doses higher than or lower than 41.5 Gy may be less compatible with wound healing research. The 30.4 Gy dose caused mild injuries which had the capacity to resolve as early as 20 days post-irradiation without intervention. Studying wounds which do not last long enough to allow a treatment to exert its effect could result in a failure to detect an existing therapeutic benefit. At 52.6 Gy and higher doses, early contraction could complicate wound analysis and higher doses may cause excessive insult to cell viability. Wounds in higher dose groups contained moist desquamation centrally, while scabbing and crusting were limited to margins. Higher doses may have caused extensive destruction to epidermal basal cells decreasing cellular function within borders, causing a breakdown in skin integrity which permitted fluid to traverse barriers and produce moist desquamation. In the 30.4 Gy and 41.5 Gy groups, cellular function may have persisted to a degree sufficient to allow crusting and other signs of underlying healing processes to occur within central wound areas.

Moderate doses may allow viable cells to remain so that restorative therapies can produce an observable benefit. However, if cellular function is eliminated beyond recovery, demonstrating the efficacy of treatments which revitalize moderately depleted or weakened cell populations may be elusive. Withers et al. [5] developed a technique to assess viability of irradiated cells in vivo, this method may be helpful to illustrate the relationship between x-ray dose and the extent of cell survival in athymic rats.

As dose was increased, contraction began sooner and occurred to a greater extent, with striking differences observed between the 65.5 Gy and 76.5 Gy groups, as seen in Figure 3C. Contraction in the 76.5 Gy dose caused profound distortion in the shape and size of the total irradiated area and its ulcerated subset. Ulceration in this group resolved more quickly than in the 65.5 Gy dose, likely a result of reduction in the ulcerated area by means of contraction-related shrinking of the overall irradiated zone. As the size of the treated zone was reduced, some of the ulcerated skin area within was eliminated. Rather than healing, the damaged skin’s area decreased, it was not replaced with healthy tissue. Contraction in the 65.5 Gy and 52.6 Gy groups appeared later and was less extreme. The 41.5 Gy group showed no visible contraction until after the 80th day, and no contraction occurred in the 30.4 Gy group.

In the absence of contraction, the relationship among ImageJ-based ulceration percentages reflects the relationship among the true areas of the ulcerated skin zones. This proportional relationship is founded on irradiation of equal-sized skin patches and can be maintained only if the relative size and shape of treated areas remains consistent. If contraction distorts an irradiated area, the ImageJ-calculated ulceration percentage, i.e., assessing the ratio of ulcerated area and total irradiated area, becomes futile. Therefore, avoiding contraction is of critical importance when this analysis is used as an endpoint. The 76.5 Gy dose caused marked contraction, so the ulceration percentage from this group was of little value.

The current study provides a preclinical model to study late effects or various levels of radiation damage, which could also be modified to incorporate fractionated dosing. By building on the general patterns of dosage and healing described in this study, researchers may individualize their protocol to create wounds suitable for their specific aims. The next step envisioned for this model system is to explore the ability of human lipoaspirate grafts and adipose-derived stem cell-enriched grafts to improve healing in acute radiation injuries.

## Competing interests

None of the authors have any conflicts of interest associated with the work presented in this manuscript.
